# The effect of perceived relative deprivation in leader-member exchange on flow experience: the serial mediating role of perceived relative deprivation in coworker exchange and job-related anxiety

**DOI:** 10.3389/fpsyg.2025.1685263

**Published:** 2026-01-12

**Authors:** Şeyda Nur Seçkin, Cahit Çağlın

**Affiliations:** 1 Department of Business, Faculty of Economics and Administrative Sciences, İnönü University, Malatya, Türkiye; 2 Department of Marketing and Foreign Trade, Silopi Vocational School, Şırnak University, Şırnak, Türkiye

**Keywords:** perceived relative deprivation, job-related anxiety, flow at work, leader-member exchange, coworker exchange

## Abstract

**Introduction:**

Flow, first conceptualized by Csikszentmihalyi, refers to a state of deep engagement and intrinsic enjoyment in an activity. Within organizational settings, flow at work has been associated with enhanced productivity, creativity, motivation, and job satisfaction. However, while previous studies have focused primarily on the factors that facilitate flow, research examining interpersonal or relational barriers remains scarce. Drawing on Relative Deprivation Theory, this study explores whether perceived relative deprivation in leader-member interactions hinders the experience of flow at work and whether perceived relative deprivation in coworker interactions and job-related anxiety mediate this relationship among nurses-a profession characterized by high task demands and strong interdependence.

**Methods:**

A cross-sectional survey design was employed, with data collected from 406 nurses. Validated instruments measured perceived relative deprivation in leader-member and coworker interactions, job-related anxiety, and flow at work. Mediation analyses were conducted to examine direct and indirect pathways between the variables.

**Results:**

Perceived relative deprivation in leader-member interactions did not have a direct effect on flow at work. However, higher perceived relative deprivation in leader-member interactions predicted greater perceived relative deprivation in coworker interactions, which subsequently increased job-related anxiety and reduced flow at work.

**Discussion:**

The findings suggest that relational deprivation in the workplace indirectly diminishes flow by elevating job-related anxiety. These results underscore the importance of fostering equitable and supportive leader-member relationships and cohesive coworker dynamics to promote optimal psychological engagement and well-being among nurses.

## 1 Introduction

Research on flow experiences began with Mihaly Csikszentmihalyi’s pioneering studies, which aimed to examine the motivations behind individuals’ deep engagement in activities that do not appear to provide extrinsic rewards (Harris et al., 2023). It has been identified that behind individuals’ profound commitment to activities of interest lies a common experience known as an autotelic experience or flow, which can be described as a state of being fully immersed in the moment, akin to a feather drifting effortlessly with the wind (Csikszentmihalyi and Asakawa, 2016). As research progressed, it became evident that this experience is not limited to activities related to sports or the arts but can also emerge in various professions and even in routine jobs, leading to a rapid increase in studies on the subject (Csikszentmihalyi et al., 2017).

Flow at work is defined as a short-term positive experience in which an individual becomes deeply focused on work tasks, is intrinsically motivated, and feels a sense of happiness (Bakker, 2005, 2008). As a desirable experience for both employees and organizations, flow at work enables employees to effectively utilize their potential, thereby enhancing their job satisfaction and performance (Köse, 2024). Additionally, employees who experience flow at work tend to exhibit higher levels of positive affect, greater creativity, increased job engagement, and higher overall life satisfaction (Liu et al., 2023). Given the positive effects of flow at work on employee wellbeing and work outcomes, it becomes crucial to identify the conditions and factors that influence this experience within workplace environments.

The findings of a meta-analysis conducted by Liu et al. (2023) indicate that the primary factors positively influencing flow at work include proactive work behaviors, job resources (such as job autonomy and social support), certain personality traits (such as self-discipline, extraversion, and agreeableness), and specific leadership styles (e.g., authentic and transformational leadership). In contrast, research on factors that hinder flow at work remains relatively limited. Notably, there is a need for further studies examining how interpersonal relationship-based factors in workplace environments affect the experience of flow at work (Csikszentmihalyi et al., 2017).

In this study, drawing upon the Relative Deprivation Theory (Crosby, 1976, 1984), the Flow Channel Model (Csikszentmihalyi, 1975/2000), Attentional Control Theory (Eysenck et al., 2007) and previous research findings, we examine whether perceived relative deprivation in leader-member interactions constitutes a potential barrier to employees’ experience of flow at work. Employees who perceive relative deprivation due to low-quality relationships with their leaders may have limited access to resources, support, and opportunities necessary to meet job demands and achieve their career goals (Bolino and Turnley, 2009). Additionally, they may encounter obstacles in fulfilling their fundamental psychological needs in the workplace (Graves and Luciano, 2013), which could, in turn, increase their level of job-related anxiety while reducing the likelihood of experiencing flow at work.

Furthermore, the study hypothesized that as perceived relative deprivation in leader-member interactions increases, the quality of individuals’ interactions with their coworkers may deteriorate, leading to higher levels of job-related anxiety and a lower likelihood of experiencing flow at work. In other words, the sequential mediating roles of perceived relative deprivation in coworker interactions and job-related anxiety were tested in the relationship between perceived relative deprivation in leader-member interactions and flow at work. Previous studies have shown that employees with low-quality leader-member exchanges tend to exhibit negative attitudes and behaviors toward their coworkers, evaluate the work environment more negatively, and are sometimes labeled by others as socially risky individuals due to their poor relationships with their leaders (Herrero and Bornay-Barrachina, 2024; Kim et al., 2010; Lau and Liden, 2008; Sias and Jablin, 1995). Employees who experience relative deprivation as a result of low-quality relationships with their leaders may also perceive relative deprivation in their interactions with coworkers. Consequently, they may feel deprived of essential job resources-such as social support, assistance, and constructive feedback-thereby increasing their levels of job-related anxiety. Elevated job-related anxiety, in turn, is expected to impair employees’ ability to effectively use their cognitive capacities (Eysenck et al., 2007) and to deplete their emotional and motivational resources (McCarthy et al., 2016; Prem et al., 2016), ultimately weakening their flow experience at work.

The present study is expected to contribute to the relevant field in several ways. Research on flow in the field of management and organization is still in its developmental stage (Feng, 2024), highlighting the need for further investigation. Additionally, studies examining the factors influencing flow at work have predominantly focused on identifying conditions that facilitate flow, whereas research investigating the factors that hinder this experience remains relatively scarce (Csikszentmihalyi et al., 2017). By testing whether perceived relative deprivation in leader-member interactions serves as a barrier to employees’ experience of flow at work, this study aims to expand the existing body of knowledge on the topic. Moreover, research examining how perceived relative deprivation stemming from low-quality leader and peer interactions impacts employees’ work attitudes and behaviors is highly limited (Cho et al., 2014). The findings of this study may provide valuable insights into whether perceived relative deprivation resulting from unsatisfactory interpersonal relationships in the workplace increases employees’ job-related anxiety and serves as an obstacle to their experience of flow at work, thereby contributing to the relatively limited knowledge base in this area.

The present study was conducted among nurses, a professional group that provides a meaningful context for examining the relationships proposed in this research. Nursing involves demanding work conditions characterized by high task intensity, strong interdependence among employees, and substantial emotional labor. Effective performance in such environments depends heavily on the quality of nurses’ relationships with their supervisors and coworkers (Marcomini et al., 2024; Van Giersbergen and Yoltay, 2022; Ylitörmänen et al., 2019). Although numerous studies have examined flow, research investigating factors that hinder flow at work remains relatively scarce, particularly regarding interpersonal and situational inhibitors (Csikszentmihalyi et al., 2017). Previous research has demonstrated that high-quality leader-member and team-member exchanges, along with perceived organizational and coworker support, enhance nurses’ job satisfaction, commitment, and motivation (Kim and Yi, 2019; Rodwell et al., 2017; Trybou et al., 2014). Yet, the extent to which perceived relative deprivation within these workplace relationships shapes nurses’ anxiety and flow experiences remains underexplored. Accordingly, the findings of this study are expected to contribute to a deeper understanding of the relational dynamics that shape nurses’ flow experiences at work and to offer practical insights for strengthening leadership and team interactions within healthcare organizations.

## 2 Theoretical frameworks

### 2.1 Flow at work

Flow at work is a short-term, cognitive, and emotional experience that consists of three dimensions: work engagement, enjoyment of work, and intrinsic work motivation (Bakker, 2005, 2008). The work engagement dimension can be defined as employees becoming fully immersed in their tasks to the extent that they lose their sense of time. The enjoyment of work dimension reflects the pleasure and happiness employees derive from their jobs, whereas the intrinsic work motivation dimension refers to employees’ participation in work-related activities due to the inherent satisfaction and joy these activities provide (Bakker, 2008). During the flow experience, employees can focus deeply on their work, feeling a sense of control over their tasks, intrinsic motivation, and overall happiness. Furthermore, as the flow experience at work increases, employees’ post-work energy levels have been observed to rise (Demerouti et al., 2012).

The flow experience emerges when individuals perceive their tasks as highly challenging while simultaneously believing that they possess the necessary skills and competencies to meet these challenges (Csikszentmihalyi et al., 2017). The conditions that facilitate the frequent occurrence of flow-namely, high challenge and high skill/competence-are predominantly found in the workplace or structured leisure activities. As a result, flow tends to be experienced more frequently and intensely in these contexts compared to other aspects of daily life (Moneta, 2004).

Research findings on the antecedents of flow at work indicate that stimulating job demands (Van Oortmerssen et al., 2020); perceived job autonomy and support (Liu et al., 2023); social capital, an innovative learning climate (Fagerlind et al., 2013); and psychological empowerment practices (Lan et al., 2017) positively influence the experience of flow in the workplace. Additionally, professional development opportunities at work serve as a significant source of motivation for employees and enhance the experience of flow at work (Bakker, 2008). Furthermore, individuals with an autotelic personality (Moneta, 2004) and employees with high levels of self-discipline, extraversion, and agreeableness tend to experience flow at work more frequently (Liu et al., 2023). On the other hand, unfinished tasks (Peifer et al., 2020), hindering job demands (Van Oortmerssen et al., 2020), perceived job insecurity (Basyouni and El Keshky, 2021), and work-related emotional rumination (Feng, 2024) reduce the likelihood of experiencing flow at work.

Bakker and Van Woerkom (2017) argue that the flow experience is not merely a passive state shaped by external conditions; rather, employees can actively enhance their likelihood of experiencing flow at work by consciously employing certain individual strategies. These strategies include job crafting, self-leadership, fun work design, and identifying and utilizing personal strengths. Additionally, Bakker and Van Woerkom (2017) suggest that personal resources (such as self-efficacy, optimism, and resilience) and certain organizational factors (including challenging job demands, satisfying job resources, transformational leadership, and supportive human resource practices) can enhance the effectiveness of these strategies. Indeed, a meta-analysis conducted by Liu et al. (2023) further supports this notion, demonstrating that proactive work behaviors (e.g., job crafting) are significant predictors of work-related flow and that employees with certain personal resources (e.g., self-efficacy, optimism, and emotional intelligence) are more likely to experience flow at work.

### 2.2 Job-related anxiety

Job-related anxiety refers to the feelings of uneasiness or tension that employees experience when carrying out their assigned tasks in the workplace (Mao et al., 2021; McCarthy et al., 2016). Another definition describes it as a state of distress or worry triggered by work-related factors, which individuals experience either while at work or when thinking about their job (Muschalla et al., 2013). Job-related anxiety has several negative consequences for both employee wellbeing and individual and organizational productivity (Muschalla, 2016). Anxiety depletes employees’ cognitive and emotional resources, thereby reducing their performance (McCarthy et al., 2016), and may even trigger unethical behaviors (Zhang et al., 2020). Moreover, anxious employees tend to exhibit avoidance behaviors in the workplace (Muschalla, 2016) and, despite receiving organizational support, are less likely to engage in innovative work ideas and behaviors (Chouchane and St-Jean, 2023).

According to Muschalla (2016), work environments contain numerous factors that can trigger anxiety in individuals. For instance, time and performance pressure on employees, inadequate job resources, conflicts and competition among organizational members, the coercive power and control exercised by managers, unfavorable working conditions that threaten employee health-these are some of the factors that contribute to employee anxiety (Muschalla, 2016). Yip et al. (2020) further highlight organizational culture as a potential factor that can induce anxiety among employees. When organizational norms and values are not well established, when there is no consensus among organizational members regarding these norms, when employees’ personal values do not align with organizational expectations, or when the organizational culture is highly outcome-oriented, employees are more likely to feel uneasy and anxious at work (Yip et al., 2020). Additionally, workplace political activities (Haider et al., 2020), perceived psychological contract breaches (De Clercq et al., 2021), organizational exclusion, and workplace incivility (Mohsin et al., 2022) have been found to exacerbate job-related anxiety among employees.

### 2.3 Perceived relative deprivation

Relative deprivation can be defined as an individual’s perception of themselves or their social group as being disadvantaged compared to other individuals or groups, accompanied by negative emotions such as anger, resentment, and dissatisfaction (Smith et al., 2018; Walker and Pettigrew, 1984). Relative deprivation is a subjective evaluation rather than an absolute reality. In fact, it has been noted that individuals who are objectively in the most deprived conditions may not necessarily perceive themselves as disadvantaged (Crosby, 1976).

The concept of relative deprivation was first introduced by Stouffer et al. (1949) to explain the discrepancy between soldiers’ levels of job satisfaction and their objective working conditions. Research has shown that soldiers’ job attitudes are not solely determined by objective working conditions; rather, their perceptions of what they believe they deserve and their expectations significantly influence their attitudes (Feldman et al., 2002). Since its initial introduction, the concept of relative deprivation has been widely used by researchers to analyze social issues. It has become a significant topic, demonstrating its critical role in shaping both individual and collective responses to social problems across different social and temporal contexts (Feldman et al., 2002; Smith et al., 2018; Smith et al., 2012).

According to Crosby (1976) 1984, individuals’ perception of themselves as being in a state of relative deprivation is contingent upon several prerequisites. These conditions can be outlined as follows: the individual (1) observes that others possess X (any asset, condition, etc. perceived as lacking); (2) desires to have X; (3) believes they deserve X; (4) perceives X as attainable; and (5) does not hold themselves or their group responsible for not possessing X (Walker and Pettigrew, 1984). The state of relative deprivation generally arises when individuals compare their circumstances to an alternative based on the principle of “what should be.” This emphasis on “deserving” or “entitlement” differentiates relative deprivation from other psychological theories and constructs. Therefore, relative deprivation not only reflects the observation that others have more but also embodies the perception that fundamental principles of justice have been violated in the observed inequality (Smith et al., 2012; Smith and Huo, 2014).

Research examining the concept of relative deprivation within an organizational context remains limited (Cho et al., 2014). However, the findings of these few studies suggest that perceived relative deprivation is positively associated with turnover intentions (Cho et al., 2014), negatively impacts individuals’ attitudes toward their current jobs and overall careers (Feldman et al., 2002), and has detrimental effects on both mental and physical health (Buunk and Janssen, 1992).

## 3 Theoretical model

### 3.1 The impact of perceived relative deprivation in leader-member exchange on job-related anxiety and flow at work

Leader-Member Exchange theory posits that leaders, due to their limited resources such as time and energy, do not engage in exchange relationships of equal quality with all subordinates (Dansereau et al., 1975). Leaders develop high-quality relationships with certain employees, providing them with additional resources and support, while they interact with others strictly within the framework of formal, contract-based work relationships. This differentiation in leader-member interactions leads to the emergence of in-group and out-group classifications within the workplace (Graen and Uhl-Bien, 1995).

Out-group members tend to compare their current workplace situation with that of their colleagues who have higher-quality leader-member interactions (in-group members), which may lead to relative deprivation (Bolino and Turnley, 2009). This is because out-group members are often assigned routine tasks by their leaders, receive less support, and have more limited opportunities for career advancement. As a result, these employees may perceive their leader’s approach as unfair and see themselves as disadvantaged in terms of their interactions with the leader, experiencing perceived relative deprivation (Bolino and Turnley, 2009; Karacay et al., 2023; Martin et al., 2018; Van Breukelen et al., 2002).

The sense of relative deprivation stemming from differentiated leader-member exchanges negatively impacts both employees’ wellbeing and their performance. Employees who lack high-quality leader-member exchanges experience higher levels of job stress, develop negative attitudes toward the workplace, and may engage in counterproductive work behaviors, particularly when they believe that their relationship with the leader cannot be improved (Bolino and Turnley, 2009). Furthermore, as perceived relative deprivation based on leader-member interactions increases, employees’ organizational commitment weakens, their engagement in organizational citizenship behaviors declines, and they are more likely to consider leaving their jobs (Karacay et al., 2023). Additionally, research indicates that higher levels of perceived relative deprivation are associated with decreased job performance and more frequent service sabotage behaviors (Dai et al., 2016).

As perceived relative deprivation in leader-member exchange increases, employees are expected to experience higher levels of job-related anxiety and a reduced likelihood of experiencing flow at work. Employees who perceive themselves as relatively deprived due to low-quality interactions with their leaders may have limited access to support and resources necessary for achieving their work goals (Bolino and Turnley, 2009). Consequently, they may feel less autonomous and competent at work, experience weakened feelings of belonging toward their job and workplace (Graves and Luciano, 2013), and encounter obstacles that hinder their ability to experience flow at work. Additionally, negative emotions associated with relative deprivation, such as anger, resentment, frustration, and a sense of being blocked (Smith et al., 2018), may deplete employees’ cognitive and emotional resources. These emotions not only hinder employees’ ability to effectively utilize their knowledge and skills but also contribute to increased job-related anxiety while reducing their likelihood of experiencing flow at work. Based on this perspective, the following hypotheses are proposed:

*H1*: Perceived relative deprivation in leader-member exchange positively affects job-related anxiety.

*H2*: Perceived relative deprivation in leader-member exchange negatively affects flow at work.

### 3.2 The impact of perceived relative deprivation in leader-member exchange on perceived relative deprivation in coworker interactions

As relative deprivation stemming from low-quality leader-member exchange increases, employees are likely to experience heightened perceived relative deprivation in their interactions with coworkers. Research suggests that employees with lower-quality leader-member exchanges perceive lower levels of procedural and distributive justice in the workplace, feel less identified with their work teams, and are less likely to engage in altruistic behaviors at work. These employees also tend to be reluctant to provide constructive feedback, collaborate with colleagues, and share information (Bowler et al., 2010; Kim et al., 2010; Lee, 2001; Lee et al., 2022; Tse et al., 2012; Tse, 2014). Furthermore, these employees are more likely to perceive the workplace atmosphere negatively, experience more conflicts with coworkers, and evaluate their relationships with colleagues through a skeptical and pessimistic lens. This tendency is largely driven by the negative emotions associated with relative deprivation, such as anger, resentment, and jealousy (Herrero and Bornay-Barrachina, 2024).

Employees with low-quality leader-member exchange are often perceived by their colleagues as less trustworthy (Lau and Liden, 2008) and may be labeled as difficult or problematic individuals, making it risky to form friendships with them (Herrero and Bornay-Barrachina, 2024). Even if these employees wish to strengthen their relationships with coworkers, such labels can serve as significant barriers, forcing them to maintain unsatisfactory workplace relationships (Herrero and Bornay-Barrachina, 2024; Sias and Jablin, 1995). Building on the aforementioned research findings, it can be expected that employees experiencing relative deprivation in their leader-member exchange may also struggle to establish high-quality interactions with their coworkers. Believing that they do not receive the support and assistance they deserve, they may develop perceived relative deprivation in their interactions with colleagues as well.

*H3*: Perceived relative deprivation in leader-member exchange positively affects perceived relative deprivation in coworker interactions.

### 3.3 The serial mediation role of perceived relative deprivation in coworker interactions and job-related anxiety in the relationship between perceived relative deprivation in leader-member exchange and flow at work

In the relationship between perceived relative deprivation in leader-member exchange and flow at work, perceived relative deprivation in coworker interactions and job-related anxiety are expected to play a serial mediating role. According to the flow channel model (Csikszentmihalyi, 1975/2000), the key factor in employees’ ability to experience flow at work is the balance between the perceived difficulty level of tasks and the skills they believe they possess (Van Oortmerssen et al., 2020). The model suggests that when individuals perceive the level of challenge in an activity to exceed their perceived skillset, they are likely to experience worry or job-related anxiety (Nakamura and Csikszentmihalyi, 2002).

Employees who experience relative deprivation due to low-quality interactions with their leaders and coworkers may perceive job demands as more challenging and view themselves as less competent in handling these demands, which, in turn, may increase job-related anxiety. In the workplace, high-quality interpersonal relationships-with both leaders and colleagues-provide employees with essential job resources such as assistance in fulfilling their roles, collaboration, knowledge sharing, and constructive feedback, thereby strengthening them both psychologically and structurally (Chiaburu and Harrison, 2008; Graves and Luciano, 2013; Ji et al., 2023; Lan et al., 2017). Employees who perceive themselves as disadvantaged in their relationships with their leaders and colleagues may feel deprived of the support and assistance, they need at work, leading to a sense of psychological and structural disempowerment. This situation is likely to increase their perceived difficulty in handling job demands while simultaneously weakening their belief in their ability to cope with these challenges. As a result, they may experience heightened job-related anxiety. It is expected that increasing work-related anxiety will, in turn, negatively affect the three core components of the flow experience at work—absorption, intrinsic motivation, and work enjoyment. According to the Attentional Control Theory (Eysenck et al., 2007), anxiety impairs individuals’ ability to use their cognitive resources effectively. As anxiety levels rise, employees tend to divert their attention away from task-related goals toward potential threats or negative outcomes. Consequently, their cognitive capacity narrows and their ability to maintain focused attention diminishes. Employees experiencing higher levels of work-related anxiety are therefore more likely to channel their mental energy toward possible obstacles, fears of failure, or negative evaluation rather than toward their work tasks, which makes it difficult for them to sustain deep task engagement. The weakening of attentional and concentration processes disrupts absorption and the state of mindful presence that constitute the essence of the flow experience. Moreover, the constant state of vigilance, uneasiness, and fear of failure accompanying work-related anxiety tends to deplete individuals’ emotional and psychological resources, thereby increasing emotional exhaustion (McCarthy et al., 2016). In addition to cognitive resource depletion, employees may experience a loss of psychological energy, which undermines their ability to establish a positive emotional connection with their work. As a result, their sense of enjoyment and intrinsic motivation derived from work is likely to diminish. The hypothesis developed is as follows:

*H4*: Perceived relative deprivation in coworker interactions and job-related anxiety serially mediate the relationship between perceived relative deprivation in leader-member exchange and flow at work.

**FIGURE 1:**
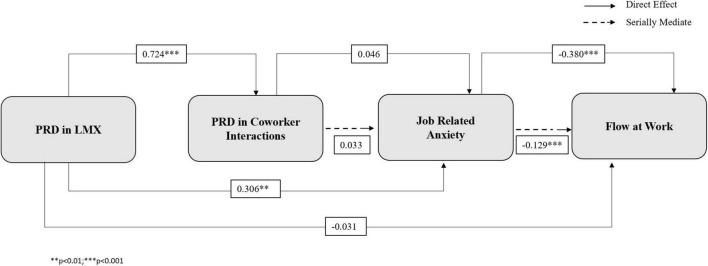
Research model. PRD in LMX, perceived relative deprivation in leader-member exchange; PRD in Coworker Interactions, Perceived relative deprivation in coworker interactions.

The research model is presented in Figure 1.

## 4 Materials and methods

### 4.1 Ethical approval

This study was conducted in accordance with the ethical approval granted by the Ethics Committee of Şırnak University, as stated in the official document dated May 2, 2023, with reference number E.66395/61864.

### 4.2 Sample

The research data were collected through a survey conducted among nurses working in state hospitals, primary healthcare centers, community and public health centers, as well as provincial and district health directorates in Şırnak. According to the information provided by the Şırnak Provincial Health Directorate, the total number of nurses in the province is 832. A total of 415 participants were reached using the convenience sampling method; however, 9 questionnaires were excluded due to incomplete or randomly filled responses, leaving 406 valid survey forms for analysis.

An a priori power analysis was conducted using G*Power 3.1 for the dependent variable Flow at Work with the independent variable PRD in LMX and the mediators (PRD in coworker interactions and Job-related anxiety) using linear multiple regression: fixed model, R^2^ deviation from zero. The analysis indicated that a minimum of 77 participants was required to detect a medium effect (*f*^2^ = 0.15) (Cohen, 1988) with α = 0.05 and 80% power. Our sample (*n* = 406) exceeded this minimum, ensuring sufficient statistical power.

### 4.3 Scales

#### 4.3.1 Perceived relative deprivation in leader-member and coworker interaction

The Perceived Relative Deprivation in Leader-Member and Coworker Interaction Scales, originally developed by Tougas et al. (2004); Cronbach’s alpha = 0.90), were adapted into Turkish by Karaçay (2014) and Özkan (2018) following a standard translation and validation procedure. First, two independent translators translated the original English items into Turkish, and an independent reviewer reconciled any differences. The Turkish items were then back-translated into English to ensure consistency with the original scale, and necessary adjustments were made. A pilot study was conducted to assess the clarity and comprehensibility of the items, and ambiguous items were revised. Subsequently, exploratory factor analysis (EFA) was performed to examine the dimensionality of the scales, and Cronbach’s alpha coefficients confirmed their reliability. An example composite item is as follows: *“1(a): I believe that, compared to other employees, I receive less appreciation from my manager. 1(b): I am not satisfied with this situation.”*

Similarly, perceived relative deprivation concerning coworkers also consists of four composite items that include pairs of cognitive and emotional statements. Participants were asked to evaluate each pair together, and the mean scores of these pairs were computed. An example composite item is: *“1(a): I have the impression that my coworkers appreciate me less compared to others. 1(b): I am not satisfied with this situation.”*

#### 4.3.2 Job-related anxiety

To assess employees’ levels of job-related anxiety, the scale developed by Parker and DeCotiis (1983; Cronbach’s alpha = 0.81) and adapted into Turkish by Gül and Koçak (2021) was employed. The scale is unidimensional and consists of five items. An example item is: *“I feel restless and tense because of my job.”*

#### 4.3.3 Flow at work

To measure the extent to which employees experience flow at work, the Work-Related Flow Inventory developed by Bakker (2008) and adapted into Turkish by Mavi et al. (2021) was used. The scale consists of three dimensions: absorption (4 items), work enjoyment (4 items), and intrinsic motivation (5 items), totaling 13 items. Example items for each dimension are as follows: Absorption, *“While performing my job, I do not think about anything else.”*; work enjoyment: *“I perform my job with great pleasure.”*; ıntrinsic motivation: *“I would still do this job even if I were paid less.”*

Bakker (2008) emphasizes that for employees to be considered as experiencing flow at work, they must score high across all components of the construct. Accordingly, researchers are encouraged to combine the three dimensions into a single overall flow score. In line with this approach, flow at work was not examined as separate dimensions in this study but rather as a unidimensional construct. Flow at Work Scale (Bakker, 2008): Cronbach’s alpha was high for Work Enjoyment (around 0.90), acceptable for Absorption (around 0.80), and satisfactory for Intrinsic Work Motivation (around 0.75).

All scales used in the study were based on a five-point Likert-type scale, with responses ranging from 1 = “Strongly Disagree” to 5 = “Strongly Agree.”

### 4.4 Findings

#### 4.4.1 Descriptive statistics of the sample

An examination of the descriptive statistics related to the sample reveals that more than half of the participants were female (58.1%), while 41.9% were male. Nearly half of the sample (48.5%) were single, whereas 51.5% were married. A significant majority (82.3%) held a bachelor’s degree, while 14.5% were high school or associate degree graduates, and 3.2% had a master’s degree. Regarding age distribution, more than half of the participants (59.9%) were under the age of 30, while 32.3% were between 30 and 35 years old. Additionally, 8.1% of the participants were between 36 and 43 years old. In terms of work experience, 30.5% of the participants had been working in their current institution for less than 3 years. The proportion of those with 3–5 years of experience was 31.6%, while 29.3% had been employed for 6–10 years. The percentage of participants with more than 10 years of work experience was 8.6%.

#### 4.4.2 Validity and reliability analysis

In the initial stage of data analysis, the construct validity, convergent validity, discriminant validity, and reliability of the scales used in the study were tested. To examine the construct validity of the scales, a confirmatory factor analysis (CFA) was conducted using the maximum likelihood estimation method and bootstrapping (*n* = 5,000) in the AMOS 23.0 software. To assess convergent and discriminant validity as well as reliability, the average variance extracted (AVE), composite reliability (CR), and Cronbach’s Alpha (α) values were calculated for each scale. The results obtained are presented in Table 1.

**TABLE 1:** Results of confirmatory factor analysis, average variance extracted (AVE), composite reliability (CR), and Cronbach’s alpha (α) values.

Variables	χ ^2^/(df)	RMSEA	TLI	GFI	CFI	SRMR	AVE	CR	α
PRD in LMX	0.921	0.000	1.00	0.999	1.00	0.0069	0.544	0.824	0.832
PRD in coworker interactions	0.560	0.000	1.00	0.999	1.00	0.0062	0.635	0.874	0.874
Job-related anxiety	0.272	0.000	1.00	1.00	1.00	0.0031	0.577	0.844	0.854
Flow at work	3.210	0.074	0.927	0.934	0.943	0.0583	0.569	0.786	0.856

Fit indices and acceptable thresholds: χ^2^/df ≤ 4–5; RMSEA = 0.06–0.08; TLI = 0.90–0.94; GFI = 0.85–0.89; CFI ≥ 0.95; SRMR < 0.08. Good Fit Index Values: χ^2^/df = ≤ 3; RMSEA = ≤ 0.05; GFI ≥ 0.90; TLI ≥ 0.95; CFI ≥ 0.97; SRMR < 0.05 (Byrne, 2016; Gürbüz, 2019; Meydan and Şeşen, 2015).

When examining the results presented in Table 1, it is observed that all scales, except for the flow at work scale, exhibit good fit index values. The fit indices for the flow at work scale also fall within acceptable thresholds. In addition, all scales used in this study have Average Variance Extracted (AVE) values above 0.50 and Composite Reliability (CR) values above 0.70. These findings indicate that the scales demonstrate high internal consistency and adequate convergent validity (Hair et al., 2010). Specifically, the CR values exceeding 0.70 confirm that the scales are reliable, supporting the robustness and credibility of the research results.

To assess whether the scales used in the study possess discriminant validity, a correlation analysis was conducted. The square roots of the average variance extracted (√AVE) for each variable were compared with the correlation coefficients between the variables (Fornell and Larcker, 1981). Additionally, the maximum squared variance (MSV) for each factor and the average shared squared variance (ASV) were calculated. The results obtained are presented in Table 2.

**TABLE 2:** Correlation analysis.

Variables	Tolerance	VIF	MSV	ASV	1	2	3	4
1. PRD in LMX	0.643	1.556	0.38	0.17	**(0.74)**	**(0.79)**		
2. PRD in coworker interactions	0.653	1.531	0.38	0.23	0.62**			
3. Job-related anxiety	0.902	1.108	0.10	0.07	0.32**	0.26**	**(0.76)**	
4. Flow at work			0.03	0.02	-0.11*	-0.04	-0.17**	**(0.75)**
Mean					2.57	2.19	2.92	3.17
Standard deviation					0.902	0.829	0.900	0.630

**p < 0.01;

*p < 0.05.

Diagonal elements in parentheses represent the square roots of the average variance extracted (√AVE) for each construct.

The findings presented in Table 2 indicate that the correlation coefficients between all variables are smaller than the square root of the average variance extracted (√AVE) for each variable. Additionally, the squared shared variance (MSV) and the average of the squared shared variances (ASV) for each variable are found to be lower than the AVE values of the respective variables. Therefore, it can be stated that the scales demonstrate discriminant validity (Fornell and Larcker, 1981; Hair et al., 2010).

Variance Inflation Factor (VIF) and Tolerance values were calculated to check for multicollinearity among the independent variables (Hair et al., 2010; Field, 2013). All VIF values were below 5, and Tolerance values were above 0.2, indicating no multicollinearity problem.

When examining the results of the correlation analysis presented in Table 2, it can be observed that perceived relative deprivation in leader-member interactions is positively related to perceived relative deprivation regarding colleagues and job-related anxiety (*r* = 0.62, *p* < 0.01; *r* = 0.32, *p* < 0.01, respectively). Additionally, a negative relationship is found between perceived relative deprivation in leader-member interactions and flow at work (*r* = −0.11, *p* < 0.05). On the other hand, a negative relationship exists between job-related anxiety and flow at work (*r* = −0.17, *p* < 0.01).

#### 4.4.3 Testing the measurement model

In the second phase of the analysis, the measurement model of the study and the alternative models were tested through confirmatory factor analysis using the maximum-likelihood and bootstrap methods (*n* = 5,000). Model 1 represents the current measurement model of the study and consists of four factors (perceived relative deprivation in leader-member interactions, perceived relative deprivation regarding colleagues, job-related anxiety, and flow experience). Model 2 is a three-factor alternative measurement model, where perceived relative deprivation regarding leader-member and colleagues is combined under a single factor. In Model 3, perceived relative deprivation in leader-member interactions, perceived relative deprivation regarding colleagues, and job-related anxiety are combined under one factor, resulting in two factors. Finally, in Model 4, all variables are grouped under a single factor. To control for common method bias, all survey items were subjected to Harman’s single-factor test. The results indicated that a single factor explained 23% of the total variance, below the recommended 50% threshold (Aguirre-Urreta and Hu, 2019), suggesting that common method bias was not a serious concern. Additionally, data were collected from multiple sources and at different times to minimize potential bias (Podsakoff et al., 2003; Podsakoff et al., 2012). The results obtained are presented in Table 3.

**TABLE 3:** Goodness-of-fit index values for alternative measurement models.

Model	χ ^2^	df	χ ^2^/df	RMSEA	TLI	GFI	CFI	SRMR	Model comparison		
										Δχ ^2^	Δ (df)
Model 1	724,876	263	2,756	0.066	0.896	0.880	0.909	0.0678	–	–	–
Model 2	929,247	267	3,480	0.078	0.853	0.841	0.869	0.0728	M2-M1	204,371	4
Model 3	1,574,245	270	5,831	0.109	0.714	0.733	0.743	0.1081	M3-M1	849,369	7
Model 4	1,834,283	271	6,769	.0.119	0.653	0.703	0.692	0.1537	M4-M1	1,109,407	8

Fit indices and acceptable thresholds: χ^2^/df ≤ 4–5; RMSEA = 0.05–0.08; TLI = 0.90–0.95; GFI = 0.85–0.89; CFI ≥ 0.90; SRMR < 0.08 (Byrne, 2016; Gürbüz, 2019, p. 34; Meydan and Şeşen, 2015, p. 37).

According to the results presented in Table 3, the goodness-of-fit index values for Model 1 (the current measurement model) fall within acceptable limits. However, the goodness-of-fit index values for Model 2, Model 3, and Model 4 are found to be outside the acceptable limits. Therefore, it was concluded that the current measurement model provides the best fit for the data set, and the data analysis proceeded using Model 1.

#### 4.4.4 Testing the structural model

**FIGURE 2:**
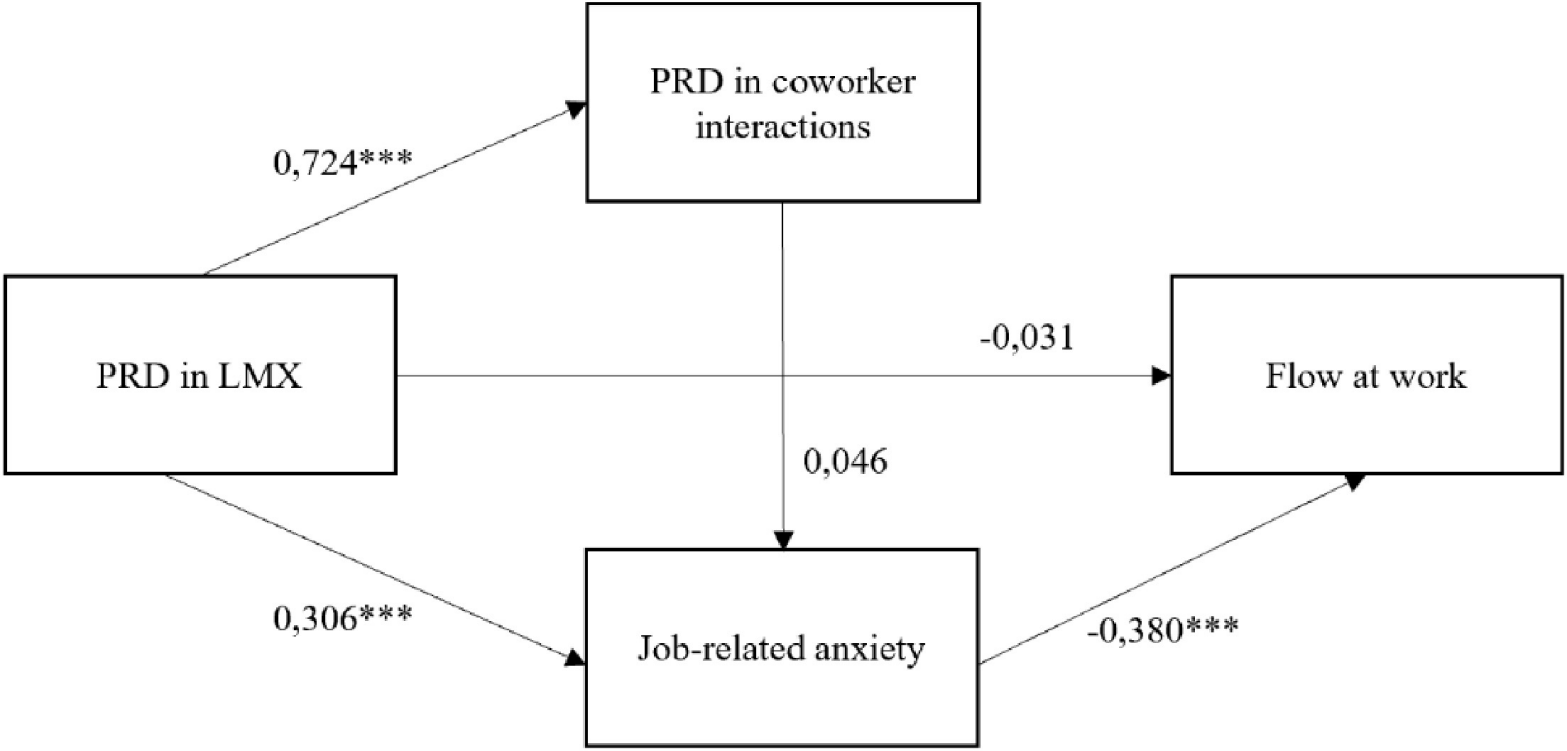
Research model. **p < 0.01; ***p < 0.001.

In the final phase of the data analysis, the structural model of the study was tested using the maximum-likelihood and bootstrap methods (*n* = 5,000). The goodness-of-fit index values for the structural model are as follows: χ^2^/(df): 2.746; RMSEA: 0.066; CFI: 0.909; TLI: 0.897; GFI: 0.879; SRMR: 0.0679. The path coefficients for the direct and indirect effects between the variables are presented in Figure 2.

When examining the results presented in Table 4, it can be observed that perceived relative deprivation in leader-member interactions positively affects job-related anxiety (β = 0.306, *p* < 0.01), but has no direct significant effect on flow at work (β = −0.031, *p* > 0.05). Therefore, Hypothesis H1 is supported, while Hypothesis H2 is rejected. Additionally, it is observed that perceived relative deprivation in leader-member interactions positively affects perceived relative deprivation regarding colleague interactions (β = 0.724, *p* < 0.001). Thus, Hypothesis H3 is supported.

**TABLE 4:** Direct and indirect effects.

Effects	Beta	SD	% 95 CI (lower–upper)
**Direct effect**			
PRD in LMX → PRD in coworker interactions	0.724***	0.049	[(0.619)–(0.811)]
PRD in LMX → Job-related anxiety	0.306**	0.100	[(0.100)–(0.505)]
PRD in LMX → Flow at work	-0.031	0.068	[(-0.165)–(0.101)]
PRD in coworker interactions → Job-related anxiety	0.046	0.100	[(-0.166)–(0.228)]
Job-related anxiety → Flow at work	-0.380***	0.070	[(-0.511)–(-0.235)]
**Indirect effect**			
PRD in LMX → PRD in coworker interactions → Job-related anxiety	0.033	0.073	[(-0.124)–(0.168)]
PRD in LMX → PRD in coworker interactions → Job-related anxiety → Flow at work	-0.129***	0.036	[(-0.211)–(-0.068)]

*p* < 0.05;

**p < 0.01;

***p < 0.001;

Beta, standardized beta coefficient; SD, standard deviation; CI, confidence interval.

When examining the indirect effects, it is observed that in the relationship between perceived relative deprivation in leader-member interactions and flow at work, perceived relative deprivation regarding colleagues and job-related anxiety play a serial mediating role. Thus, Hypothesis H4 is supported (β = −0.129, *p* < 0.001).

## 5 Conclusion

This study revealed that perceived relative deprivation in leader-member exchange does not directly affect employees’ flow at work but influences it indirectly through perceived deprivation in coworker interactions and job-related anxiety. When employees feel deprived in their relationships with leaders, this perception extends to coworkers, increases anxiety, and reduces their ability to experience flow. By integrating the Relative Deprivation Theory with the Flow Channel Model, this research advances understanding of how social comparison and perceived injustice within organizations can hinder positive psychological states such as flow. The findings emphasize that employees’ optimal work experiences depend not only on job characteristics but also on the fairness and quality of workplace relationships. Practically, managers should foster fair, transparent, and supportive interactions among both leaders and coworkers to minimize deprivation perceptions and enhance engagement. Conducted in a collectivist context, this study also suggests that interpersonal harmony and fairness may play a stronger role in predicting flow than in individualistic cultures.

## 6 Discussion

The study revealed that perceived relative deprivation in leader-member interactions significantly increased employees’ levels of job-related anxiety. According to Leader-Member Exchange (LMX) theory (Graen and Uhl-Bien, 1995), leaders distribute their limited resources unevenly among subordinates, which inevitably creates an “in-group-out-group” distinction within the organization. Employees in the out-group tend to compare themselves with those who have closer relationships with the leader. When they perceive themselves to be in a disadvantaged position, this perception generates feelings of exclusion, devaluation, and powerlessness (Bolino and Turnley, 2009). Moreover, low-quality leader-member relationships deprive employees of critical social and structural job resources and hinder the fulfillment of their basic psychological needs-autonomy, competence, and relatedness-thus triggering stress and exhaustion (Graves and Luciano, 2013; McCarthy et al., 2016). In this context, the findings suggest that as perceived relative deprivation in leader-member relationships increases, employees are likely to believe that they receive insufficient support, guidance, and feedback from their leaders. Consequently, they may feel less autonomous and competent in their jobs, experience weakened perceptions of control, and interpret their work environment as uncertain and threatening, which in turn heightens their job-related anxiety.

An unexpected yet noteworthy finding of the study was that perceived relative deprivation in leader-member interactions did not directly and significantly affect employees’ experience of flow at work. The meta-analysis by Chiaburu and Harrison (2008) indicated that employees spend a considerable portion of their social interactions with coworkers and that these peer interactions exert a stronger influence on work attitudes and behaviors compared to leader interactions. Considering the nursing context in which this study was conducted, this finding gains further significance. Nursing is a profession built upon human relations (Durmuş and Yıldırım, 2018), and the successful execution of duties largely depends on cooperation, communication, and information exchange among colleagues (Van Giersbergen and Yoltay, 2022). Nurses, more than most other occupational groups, rely on the informational, emotional, and practical support of their coworkers, meaning that their work attitudes and behaviors are highly influenced by the quality of peer collaboration and interaction (Marcomini et al., 2024; Ylitörmänen et al., 2019). Therefore, the absence of a direct relationship between perceived relative deprivation in leader-member interactions and flow experience among nurses can be interpreted as evidence that this effect operates indirectly-through coworker relationships and job-related anxiety.

Another key finding of the study was that as perceived relative deprivation in leader-member interactions increased, so did employees’ perceptions of relative deprivation in coworker interactions. This result suggests that employees who feel deprived due to low-quality relationships with their leaders also tend to perceive poorer-quality relationships with their coworkers. Low-quality leader-member relationships foster perceptions of injustice, which erode trust, cooperation, and mutual support within the team (Lee, 2001; Tse et al., 2012). The deprivation experienced in leader-member interactions thus extends beyond vertical relationships and shapes horizontal relationships as well, leading employees to view those with strong leader ties as a privileged “in-group” (Martin et al., 2018). Herrero and Bornay-Barrachina (2024) similarly demonstrated that differentiation in leader-member relationships can create a contagion effect on team dynamics, where favoritism shown by the leader triggers feelings of exclusion, envy, and mistrust among other employees. Likewise, Kim et al. (2010) found that low-quality leader-member relationships reduce helping and social support behaviors; when employees perceive their coworkers as members of a “favored group,” their inclination to cooperate weakens. Taken together, these studies indicate that the in-group-out-group distinction originating in vertical relationships extends to horizontal coworker relations-a conclusion supported by the present findings.

Finally, the study confirmed the sequential mediating role of perceived relative deprivation in coworker interactions and job-related anxiety in the relationship between perceived relative deprivation in leader-member interactions and flow experience at work. Deprivation experienced in leader-member relationships creates an “us versus them” perception that undermines trust and belonging among coworkers, leading to feelings of social exclusion and relative deprivation in peer interactions (Bolino and Turnley, 2009; Karacay et al., 2023; Martin et al., 2018; Tse et al., 2012; Lee, 2001). Perceived relative deprivation among coworkers subsequently increases employees’ job-related anxiety. This result can be interpreted through the lens of the Flow Channel Model (Csikszentmihalyi, 1975/2000), which posits that anxiety arises when perceived challenges exceed perceived skills. Employees who view themselves as disadvantaged in their coworker relationships may feel isolated and deprived of the structural and social support needed to meet job demands, leading them to perceive their tasks as more difficult and threatening.

The final link in the mediation chain revealed that job-related anxiety weakened employees’ flow experiences. According to Attentional Control Theory (Eysenck et al., 2007), anxiety diverts attention from task goals toward potential threats and negative outcomes, narrows cognitive capacity, and impairs concentration. Thus, job-related anxiety stemming from perceived deprivation in leader and coworker relationships can be expected to disrupt the core component of flow-deep absorption. Furthermore, the persistent vigilance, rumination, restlessness, and fear of failure that accompany anxiety deplete not only employees’ cognitive but also their emotional and psychological resources (De Clercq et al., 2020; McCarthy et al., 2016; Prem et al., 2016). This resource loss diminishes employees’ ability to experience intrinsic enjoyment and satisfaction from their work, making it more difficult to establish an emotional connection with their tasks and ultimately pulling them away from the state of flow.

Taken together, the findings demonstrate that perceived relative deprivation in leader-member relationships indirectly shapes employees’ work experiences. Relative deprivation within the leader-member relationship triggers deprivation in horizontal coworker interactions, which then increases job-related anxiety, and ultimately diminishes the experience of flow at work-creating a sequential chain of relational and emotional effects within organizations. These results gain additional significance within the context of the nursing profession. Nursing, by its nature, involves high levels of emotional labor, heavy workloads, and interdependent relationships (Marcomini et al., 2024; Ylitörmänen et al., 2019). Therefore, relational deprivation and the anxiety it triggers may make nurses particularly vulnerable to disruptions in their experience of work-related flow.

## 7 Theoretical implications

This study integrates Relative Deprivation Theory (Crosby, 1976, 1984), the Flow Channel Model (Csikszentmihalyi, 1975/2000), and the Attentional Control Theory (Eysenck et al., 2007) to examine how perceived relative deprivation in leader-member interactions influences the experience of flow at work, thereby offering several contributions to the existing literature. Previous research on flow has largely focused on the individual and organizational factors that facilitate flow, whereas the relational and emotional conditions that may hinder flow experiences in the workplace have received far less attention (Csikszentmihalyi et al., 2017). By investigating how low-quality interpersonal relationships and the negative emotions arising from such relationships affect employees’ flow experiences, this study expands the current understanding of the antecedents that can obstruct flow in organizational settings.

Second, the study considers relative deprivation in both vertical (leader-member) and horizontal (coworker) relationships, revealing that relational dynamics within organizations are interconnected and collectively shape employees’ psychological experiences. The findings show that relative deprivation in leader-member exchanges not only undermines vertical relationships but also triggers a cascading sense of deprivation in coworker interactions. In this respect, the study contributes to the limited body of knowledge by highlighting that relative deprivation can extend beyond leader-follower dyads and permeate other social relationships within organizations.

Third, the study demonstrates that job-related anxiety is nourished by relational deprivation and, through the loss of cognitive and emotional resources, hinders the experience of flow. In doing so, the findings provide empirical support for the Flow Channel Model (Csikszentmihalyi, 1975/2000) within an organizational context. Moreover, identifying job-related anxiety as a mediating mechanism that originates from relational deprivation and weakens flow underscores that flow is a fragile internal state-sensitive to the quality of both vertical and horizontal relationships in the workplace.

## 8 Practical implications

The findings of this study indicate that relative deprivation based on leader-member interactions functions as an indirect barrier to employees’ experience of flow at work. Therefore, it is essential to prevent the formation of relative deprivation perceptions among employees who do not have high-quality relationships with their leaders. Given that leaders possess limited time and energy, it is unrealistic to expect them to maintain equally close relationships with all subordinates (Dansereau et al., 1975). However, if the differentiation in relationship quality is grounded in explicit and transparent criteria and these criteria are clearly communicated to employees, perceptions of relative deprivation can be minimized. Contextual and task performance goals explicitly defined by the leader may serve as concrete benchmarks to justify differences in the distribution of support and resources among subordinates.

Moreover, leaders’ efforts to ensure fairness in their interactions, provide opportunities for out-group members to become part of the in-group, and maintain open, transparent, and respectful communication with all subordinates can effectively reduce perceptions of relative deprivation. In professions such as nursing-where work requires high levels of coordination, emotional labor, and mutual interdependence-leaders who adopt a fair, supportive, and transparent communication style play a vital role in sustaining employees’ work energy and intrinsic motivation. Furthermore, leader-subordinate relationships built on mutual trust can positively influence not only employee wellbeing but also patient safety and the overall quality of care.

## 9 Limitations and future research directions

As with any study, this research has several limitations. First, due to its cross-sectional and correlational design, causal relationships between the variables cannot be established. Future research employing longitudinal or experimental designs would provide stronger evidence regarding the directionality and causality of these relationships.

Another limitation concerns the single-source and single-time-point data collection, which may raise the potential issue of common method bias (Podsakoff et al., 2003). To minimize this risk, several procedural remedies were implemented (Podsakoff et al., 2003; Podsakoff et al., 2012). Participants were assured of the voluntary and anonymous nature of their participation, informed that they could withdraw at any time, and told that their responses would be analyzed in aggregate form. In addition, to test whether common method bias was a concern, both Harman’s single-factor test and a confirmatory factor analysis (CFA) were conducted by loading all items onto a single latent factor. The results indicated that common method bias was not a significant issue in this study.

A further limitation stems from the contextual scope of the research. The study was conducted in the collectivist cultural context of Turkey and was limited to a single occupational group-nurses. Therefore, the findings may not be generalizable to other professions or cultural settings. Additionally, some demographic characteristics of the sample represent another constraint. More than half of the participants were under the age of 30 (59.9%) and had been employed in their current institutions for no longer than 5 years (62.1%). Consequently, the findings may primarily reflect the experiences of younger and less tenured employees. Future research could examine whether the effects of relational deprivation on job-related anxiety and flow differ across employees of varying ages and tenure levels.

Future studies should also explore additional mediating mechanisms that may explain the link between leader-member-related deprivation and flow experience. For instance, employees who perceive relative deprivation in leader-member exchanges might interpret their poor-quality relationships with the leader as a signal of organizational injustice or obstruction, which could intensify anxiety and tension, thereby preventing the experience of flow at work.

Moreover, relative deprivation could be examined not only at the individual level, as in this study, but also at the group level. Wan et al. (2023) suggest that while individual-level deprivation tends to elicit negative emotions such as envy, hostility, and stress; group-level deprivation, when grounded in a shared sense of disadvantage, can foster solidarity, collective coping, and shared goal orientation. Accordingly, future studies could investigate whether group-level deprivation enhances team cohesion and cooperation, thereby positively influencing employees’ experience of flow at work.

Finally, since the study was conducted in a single province of Türkiye within a collectivist cultural context and focused exclusively on nurses, the findings may not be directly generalizable to other occupational groups or different cultural settings. Future research should consider incorporating individual characteristics-such as neuroticism, positive/negative affectivity, self-efficacy, perceived occupational competence, and emotion regulation strategies-as potential moderators in the research model. Prior studies indicate that anxiety does not affect all individuals equally and that personality traits influence how people perceive and manage anxiety (Bandura, 1993; Cheng and McCarthy, 2018). Bandura (1993) emphasized that self-efficacy shapes individuals’ interpretations of environmental threats and their capacity to cope with anxiety and stress; individuals with high self-efficacy perceive challenging situations as less threatening. Similarly, the Workplace Anxiety Theory (WAT) developed by Cheng and McCarthy (2018) posits that individual differences can buffer the negative impact of anxiety on performance and, under certain conditions, even enable anxiety to play a motivating role. In addition, Zhang et al. (2022) found that employees who employ cognitive reappraisal strategies manage job-related anxiety more effectively, thereby maintaining their focus and motivation. Future research examining the moderating effects of these individual characteristics on the relationships between relative deprivation, anxiety, and flow could enhance the explanatory power and generalizability of the model.

## Data Availability

The datasets presented in this article are not readily available because the dataset used in this study contains sensitive information from human participants (nurses), and full public access is restricted to protect their privacy. However, anonymized data may be made available upon reasonable request to the corresponding author.
